# Thyroid Autoimmunity Impairs Oocyte Maturation, Fertilization, and Embryo Development in Assisted Reproductive Technology in Euthyroid Infertile Patients

**DOI:** 10.3390/jcm14103385

**Published:** 2025-05-13

**Authors:** Tina Sušanj Šepić, Kristina Čavlović, Sanja Dević Pavlić, Nataša Smajla, Alenka Višnić, Anđelka Radojčić Badovinac, Neda Smiljan Severinski

**Affiliations:** 1 Department of Human Reproduction, Clinic for Gynecology and Obstetrics, University Hospital Centre Rijeka, 51000 Rijeka, Croatia; tina.susanj@gmail.com (T.S.Š.); kristina.cavlovic2@gmail.com (K.Č.); nsmajla@gmail.com (N.S.); alenka.visnic@hotmail.com (A.V.); nedass@medri.uniri.hr (N.S.S.); 2 Department of Medical Biology and Genetics, Faculty of Medicine, University of Rijeka, 51000 Rijeka, Croatia; 3 Faculty of Biotechnology and Drug Development, University of Rijeka, 51000 Rijeka, Croatia; andjelka@uniri.hr

**Keywords:** controlled ovarian hyperstimulation, embryo development, fertilization rate, in vitro fertilization (IVF), ovarian reserve, thyroid autoimmunity

## Abstract

**Background**: Thyroid autoimmunity (TAI) has been widely associated with reduced fertility; however, its impact on assisted reproductive technology (ART) outcomes in euthyroid women remains controversial. Ovarian reserve (OR) and anti-Müllerian hormone (AMH) are considered to be the most reliable predictors of controlled ovarian hyperstimulation (COH) and ART outcome. This study aims to evaluate whether TAI affects COH outcomes depending on the OR, or if TAI is an independent negative factor affecting COH outcomes. **Methods**: This study includes 341 infertile euthyroid participants under 38 years old undergoing ART at a single reproductive medicine center. The serum concentrations of sex hormones, thyrotropin (TSH), AMH, and antithyroid antibodies (ATAbs) were measured before COH. Ovarian response to COH, assessed by oocyte number and maturation (percentage of mature MII oocytes), fertilization rate (FR), and early embryo development (cleavage and blastocyst rate), were assessed in 191 participants with TAI and 150 TAI negative age-matched controls with normal ORs. The TAI group was further divided into two subgroups: the TAI1 group with normal OR (n = 120) and the TAI2 group with diminished ORs (n = 71). **Results**: The mean of the retrieved oocytes was significantly lower in TAI1 (*p* = 0.015) and expectedly significantly lower in TAI2 (*p* < 0.001) compared to the control. The percentage of MII oocytes was significantly lower in the TAI1 (*p* < 0.001) and TAI2 (*p* = 0.009) groups compared to the control group. We observed significantly lower FR (*p* = 0.002), cleavage rate (*p* = 0.020), and blastocyst rate (*p* < 0.001) in the TAI1 group compared to control. In the TAI2 group, there was a lower cleavage rate (*p* < 0.001) and blastocyst rate (*p* < 0.001) compared to the control. There was no difference in the mean percentage of MII oocytes, FR, and cleavage rate between the TAI1 and TAI2 groups, but the blastocyst rate was significantly lower (*p* < 0.001) in the TAI2 group. **Conclusions**: TAI may represent a negative predictor of in vitro fertilization outcomes by impairing oocyte maturation, fertilization rate, and embryo development in ART cycles, regardless of ORs.

## 1. Introduction

A clear pathophysiological relationship between TAI to infertility and reproductive outcomes after spontaneous conception or ART has not been fully identified. Several studies and potential pathophysiological mechanisms have been proposed to discover the pathophysiological mechanisms and the role of TAI in reproductive failure [1,2,3,4]. Researchers have hypothesized the direct infiltration of reproductive organs by thyroid antibodies, the coexistence of TAI with other autoimmune diseases, immune dysfunction leading to the inhibition of immune tolerance, and relative thyroid hormone deficiency [2,4]. TAI may be a peripheral marker of a systemic immune imbalance influencing fertilization, implantation, and pregnancy maintenance [2,4]. Notably, antithyroid antibodies (ATAbs) in the serum of infertile patients are significantly higher compared to those of fertile women of similar age, and their elevated serum concentrations have been linked to adverse pregnancy outcomes, reducing live birth rates after both spontaneous reproduction and infertility treatment [1,4,5,6,7,8].

The prevalence of TAI is up to 20% in infertile women [5]. Considering the higher prevalence of TAI among women undergoing infertility treatment, some researchers indicate the importance of screening for autoimmune thyroid dysfunction in the infertile population [3]. Therefore, ATAb screening has recently been included in initial diagnostic recommendations for infertile women [9]. While substantial evidence suggests that TAI negatively impacts reproductive outcomes, even in euthyroid patients, multiple clinical studies, systematic reviews, and meta-analyses remain inconclusive about its impacts on ART outcomes [2,3,4,5,6,7,8,9]. Discrepancies across studies may arise from differences in sample size, study design, indications for infertility treatment, type of ART procedure, definition of euthyroidism, and research objectives [10,11,12,13,14,15,16,17,18,19,20,21]. The significance of ATAbs in ART has been debated for almost three decades. Some studies report a clear negative impact of elevated ATAbs on ART outcomes [10,11,12,13,14,15,16], while others debate these findings [17,18,19,20,21]. Vissenberg et al. concluded that the presence of TPOAbs negatively affects folliculogenesis, spermatogenesis, fertilization rate (FR), and embryo quality [1], while Safarian et al. revealed a higher prevalence of suboptimal response to ovarian stimulation in TAI-positive women, a lower proportion of MII oocytes in women with TAI, lower FR, and fewer high-quality embryos [12]. On the contrary, Busnelli et al. state that TAI does not impact ART outcomes regarding the number of retrieved oocytes and fertilization [20].

One hypothesis proposes a potential immune mechanism of infertility through TPOAbs cross-reaction with hCG receptors in the oocyte’s zona pellucida. Supporting this, Monteleone et al. identified thyroid peroxidase (TPO) expression on the granulosa cells within the follicular fluid of infertile women, suggesting a direct impact of TPOAbs at the ovarian level, potentially compromising oocyte quality [22]. This immune-mediated mechanism may contribute to an unfavorable follicular environment independent of thyroid hormone levels, particularly in the early stages of autoimmunity [2,4,22]. They may generate an inflammatory response that alters the environment of the maturing oocyte, affecting ovarian reserve and embryo quality [4]. It is conceivable that the zona pellucida, which plays an essential function in the interaction between the oocyte and sperm, could be a target of ATAbs [22].

Ovarian reserve is a significant factor in the course and outcome of controlled ovarian hyperstimulation (COH), with both AMH levels and antral follicle count (AFC) being widely accepted as reliable and accurate markers. However, serum AMH measurement offers several advantages over AFC, including reduced operator dependency, lower inter-cycle variability, and broader applicability across diverse clinical settings [6,23]. Several studies suggest an association between TAI and reduced OR [24,25,26,27]. Although autoimmune disease often affects multiple endocrine glands due to shared antigenic proteins, some researchers argue that autoimmune damage of the ovary is not a common cause of reduced ovarian function [28,29]. A clear connection between TAI and OR has not been confirmed. TAI has been associated with poorer embryo quality [10,11,12], lower clinical pregnancy rates, a higher risk of miscarriage, and lower live birth rates in women treated with ART [2,7,8,14,15]. However, conflicting findings from several comprehensive reviews and meta-analyses [18,19,20] challenge the significance of these associations, and no adverse effect of TAI on ART outcomes has been confirmed. Two recent studies emphasize a potential connection between TAI, diminished OR, reduced embryo quality, and impaired developmental potential of embryos in euthyroid infertile women with reduced ORs [30,31]. Zhang et al. concluded that TAI and higher ATAb concentrations were associated with fewer retrieved oocytes and poorer embryo quality in euthyroid infertile women with diminished ORs [30]. On the other hand, the study by Magri et al. points out that TAI in women with preserved ORs, assessed using serum AMH values, had a negative effect on COH in terms of the number of MII oocytes [16].

Given these uncertainties, our study aims to investigate whether TAI affects COH outcomes, particularly in terms of oocyte maturation and embryo development, independently of ORs. By addressing this gap, our study offers a novel perspective on the impact of TAI on ART outcomes.

## 2. Materials and Methods

### 2.1. Study Design and Population

This study includes 341 euthyroid female participants under the age of 38 who were treated for infertility with COH and in vitro fertilization at the Department for Human Reproduction in Clinical Hospital Center Rijeka in the period from 2018 to 2024. Indications for IVF were categorized according to the WHO International Classification of Diseases-ICD-10. All included subjects were treated due to male, tubal, or infertility of other origin with no significant difference in the prevalence of infertility etiologies.

Serum concentrations of follicle-stimulating hormone (FSH), luteinizing hormone (LH), anti-Müllerian hormone (AMH), estradiol (E2), thyrotropin (TSH), thyroid peroxidase antibodies (TPOAbs), and thyroglobulin antibodies (TgAbs) were determined in all subjects.

The study participants were grouped into TAI-positive (n = 191) and TAI-negative groups (n = 150) based on the serum concentration of TPOAbs and TgAbs. The participants with the TPOAbs concentration ≥ 60 IU/mL and/or TgAbs ≥ 4.5 IU/mL were included as TAI-positive subjects, while all the other participants were considered TAI-negative. In the control group, all participants were TAI-negative with normal ORs (TAI0). Furthermore, TAI-positive participants were grouped according to OR defined by the serum AMH concentration into TAI1 subgroup (n = 120) with normal OR (AMH ≥ 1.1 ng/mL) and TAI2 subgroup (n = 71) with reduced OR (AMH < 1.1 ng/mL) [32]. The prevalence of TPOAbs in the TAI1 group was 90% and in the TAI2 group 77.1%. The prevalence of TgAbs in TAI1 group was 81,4% and in TAI2 69.1%.

Subjects diagnosed with subclinical thyroid dysfunction (elevated TSH, normal serum concentrations of fT3, fT4) were treated with oral levothyroxine (25–125 μg/day) before the start of ovarian stimulation until TSH levels normalized (TSH 0.55–4.20 mIU/L) according to the current recommendations [9,33]. All cycles, including the first one, were included in the analysis after the TSH levels were normalized.

Exclusion criteria included manifest hypothyroidism, diagnosed associated autoimmune diseases affecting fertility, previously diagnosed endometriosis, and age over 38 years.

For all subjects, the number of retrieved oocytes after standard COH, the proportion of metaphase II (MII) oocytes retrieved after follicle aspiration, the mean number of fertilized oocytes, and fertilization rate (FR) after conventional in vitro fertilization (IVF) or intracytoplasmic sperm injection (ICSI) method were analyzed. The IVF/ICSI ratio was approximately 1:1 in all study groups. In the control group, the percentage of ICSI was 57.7%, in the TAI1, the percentage was 51.3%, and in the TAI2, 63.6%. Embryo development was assessed by evaluating the cleavage and blastulation rates [34]. To determine the response to COH in terms of embryo viability, the percentage of developed embryos on Day 3 (68 ± 1 h) and the percentage of growing blastocysts on Day 5 and/or 6 (116 ± 2 h and/or 140 ± 2 h) post-fertilization were calculated based on the number of MII oocytes that underwent fertilization. Due to national legislation, a maximum of 12 oocytes per cycle was fertilized, while supernumerary oocytes were cryopreserved for future IVF treatments.

### 2.2. Serum Assays

Concentrations of FSH, LH, E2, and AMH were determined on the second or third day of the menstrual cycle using the electro-chemiluminescent immunochemical method (ECLIA) on the Roche cobas 6000 e601 device (Roche Diagnostics, Mannheim, Germany). TgAbs, TPOAbs, and TSH concentrations were determined using the chemiluminescent immunochemical method (CLIA) on a Siemens Atellica IM1600 device (Siemens Healthcare Diagnostics, Dublin, Ireland).

The reference values for thyroid hormones and antibodies were TSH 0.55–4.20 mIU/mL (analytical sensitivity of 0.008 mIU/L); the upper limit of the reference range was TgAbs 4.5 IU/mL (analytical sensitivity of 0.9 IU/mL), and the TPOAbs reference range was 60 IU/mL (analytical sensitivity 20 IU/mL). The performance of these assays is validated and used for routine analysis in hospital laboratories, not only for study purposes.

### 2.3. Controlled Ovarian Stimulation Protocol

All participants started flexible antagonistic ovarian stimulation protocol on the second or third day of the menstrual cycle. Recombinant FSH (Gonal-F^®^, follitropin alfa; Merck Serono, Germany) or highly purified human menopausal gonadotropin-hMG (Menopur^®^, Human menopausal gonadotropin, Ferring, Germany) were daily injected subcutaneously. The dose of administered gonadotropin was 150–225 IU/day. A transvaginal ultrasound was used to monitor follicular and endometrial growth. When the follicles reached a mean diameter of 13–15 mm, patients started with daily injections of gonadotropin-releasing hormone (GnRH) antagonist (Cetrotide^®^ 0.25 mg/mL, Cetrorelix acetate, Merck Serono, Germany). Recombinant chorionic gonadotropin was administered (Ovitrelle^®^, 250 µg choriogonadotropin alfa, Merck Serono, Germany) on the day when the three leading follicles measured 17 mm or one follicle 18 mm in diameter. After 34–38 h, follicular aspiration (Cook’s^®^ aspiration single lumen needle) was performed under ultrasonographic guidance.

### 2.4. Fertilization and Embryo Culture

Follicular fluid was immediately evaluated for cumulus–oocyte complexes (COC) by embryologists under the stereomicroscope. Isolated COCs were transferred into a Petri dish with capacitated media and stored in a humidified CO_2_ incubator with a constant temperature value and CO_2_ concentration (37 °C and 6% CO_2_). The maturation of the oocytes prepared for conventional IVF was assessed at the time of retrieval by rapid spreading of the cumulus cells in COCs and by observing the presence of the first polar body under a 200× magnification, while the oocytes prepared for ICSI were assessed after their denudation from COCs. The oocyte was considered mature if the polar body was extruded from the oocyte cytoplasm into the perivitelline space, which confirmed the metaphase II stage (MII). The immature oocytes in the germinative vesicle (GV) stage and atretic or deformed oocytes were not used for fertilization and discarded. Fertilization was performed 36–40 h after the oocyte triggering. The fertilization method was defined based on the sperm parameters of concentration, motility, and morphology after the semen preparation.

The presence of two pronuclei was assessed 16–18 h after fertilization under the stereomicroscope. If one or more than two pronuclei were seen, the fertilization was considered abnormal, and those zygotes were discarded. Developed embryos were assessed on the second (44 ± 1 h) and third day (68 ± 1 h) after fertilization and were qualified according to morphological characteristics based on the Istanbul consensus [34]. Blastocysts were graded on the fifth and/or sixth day (116 h ± 2 h and/or 140 ± 2 h) post-fertilization according to the Gardner criteria [35].

### 2.5. Statistical Analysis

Statistical analysis was performed using R software, version 4.1.1, with the tidyverse set of add-on packages and patchwork plot composer. Descriptive statistics were expressed as a mean of a 95% confidence interval. Inferential comparisons between study groups were made using analysis of variance with a *p*-value < 0.05 considered statistically significant after post hoc adjustment. The normality of distributions and homogeneity of variances were assessed using a Shapiro–Wilk’s test, Levene’s test, and Q-Q plots of residuals. The percentages were first calculated at the individual level, and this value was further averaged at the group level (for example, fertilization rate and other individual-level percentages).

## 3. Results

### 3.1. Study Group Descriptions and Hormonal Profiles

**Figure 1:**
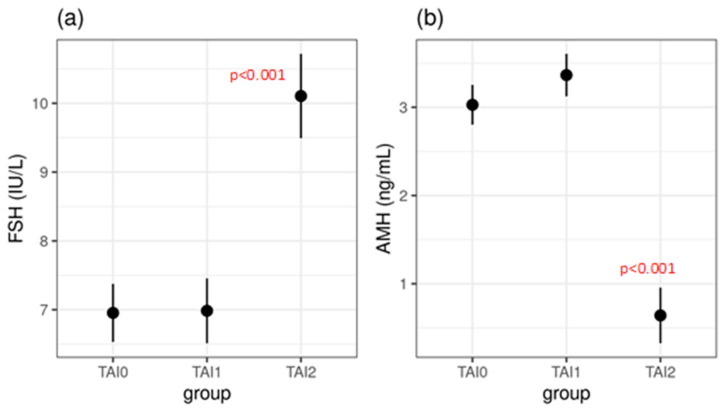
Serum FSH (**a**) and AMH (**b**) concentrations between the three investigated groups, presented as means with 95% confidence intervals. TAI0—control group, TAI1—TAI-positive participants with normal ovarian reserves, TAI2—TAI-positive participants with diminished ovarian reserves.

The mean age of the study groups was not significantly different. The hormonal status of the TAI1 subgroup was not significantly different from that of the control group, except for a significantly higher concentration of TSH (2.3 ± 1.3 vs. 1.9 ± 0.7, *p* = 0.011) (Table 1). However, the TAI2 subgroup had a different hormonal profile compared to the TAI0 and TAI1 subgroups. The results show significantly higher FSH concentration (10.1 ± 4.7 vs. 7.0 ± 1.4, *p* < 0.001 and 10.1 ± 4.7 vs. 7.0 ± 2.0, *p* < 0.001, respectively) and a significantly lower AMH concentration (0.6 ± 0.3 vs. 3.0 ± 1.3, *p* < 0.001 and 0.6 ± 0.3 vs. 3.4 ± 1.7, *p* < 0.001, respectively), which was expected and in accordance with the reduced ORs (Table 1, Figure 1).

In contrast, the average concentrations of estradiol (E2) and luteinizing hormone (LH) remained similar across all study groups. The average concentrations of TPOAbs and TGAbs did not differ significantly between the TAI1 and TAI2 groups.

### 3.2. Oocytes and Embryos

**Figure 2:**
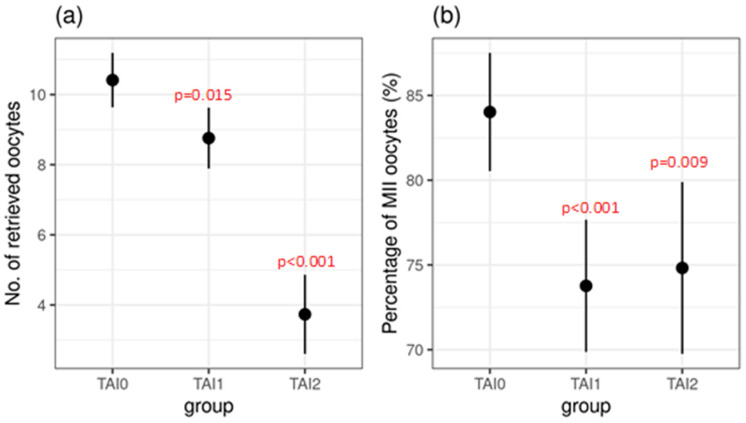
Number of oocytes retrieved (**a**) and percentage of MII oocytes (**b**), means, and 95% confidence intervals (compared to the control group). TAI0—control group, TAI1—TAI-positive participants with normal ovarian reserves, TAI2—TAI-positive participants with diminished ovarian reserves.

**Figure 3:**
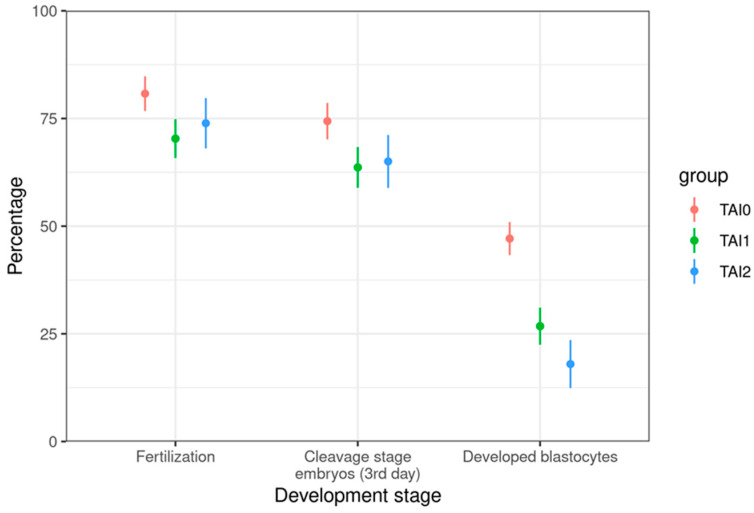
Differences in outcomes of ovarian stimulation and embryo cultures between study groups (*p*-values shown in Table 2). TAI0—control group, TAI1—TAI-positive participants with normal ovarian reserves, TAI2—TAI-positive participants with diminished ovarian reserves.

Subgroup TAI1 differed significantly from the control group and had a poorer outcomes in all of the analyzed categories: ovarian stimulation, fertilization, and embryo culture. Similarly, this was noticed in the TAI2 subgroup, except for the FR, which was not statistically different from the control group (Table 2, Figure 2 and Figure 3).

A comparison between the TAI1 and TAI2 subgroups revealed a significantly lower mean number of retrieved (8.8 ± 6.0 vs. 3.7 ± 2.5, *p* < 0.001) and fertilized oocytes (4.5 ± 2.8 vs. 2.2 ± 1.8, *p* < 0.001) in the TAI2 subgroup, reflecting differences in ORs. Moreover, the mean percentage of developed blastocysts and the blastocyst rate were significantly lower in the TAI2 group (18.0 ± 30.1 vs. 26.8 ± 25.8, *p* = 0.038 and 21.3 ± 34.7 vs. 35.7 ± 32.5, *p* < 0.002, respectively). The remaining analyzed parameters did not differ significantly between these two subgroups (Table 2).

## 4. Discussion

In our study, participants with TAI showed significantly poorer outcomes in ovarian stimulation and embryo culture compared to the control group. However, we did not observe significant differences between TAI subgroups with significantly different ORs regarding oocyte maturation, fertilization, or embryo development after ovarian stimulation. These results indicate that the poorer outcomes are more closely linked to the autoimmune condition itself rather than to OR.

While previous studies have linked TAI to a reduced OR [16,24,25,26,27], our research aimed to evaluate the impact of TAI independently of OR, specifically focusing on its effects on oocyte quality, fertilization, and embryo development. Aligning with our clinical observations on the poorer outcome of COH in women with TAI, despite normal ovarian reserve, this study aimed to confirm whether the reproductive potential is additionally impaired with TAI. Contrary to previous studies, we did not find a direct association between TAI and reduced OR, as there were no significant differences in AMH and FSH concentrations between the TAI1 subgroup and the control group. Hasegawa et al. [27] demonstrated significantly lower AMH levels in women with euthyroid TAI. However, our research did not confirm these findings, likely due to differences in study design. We stratified TAI subjects into two subgroups based on OR, and the subgroup with normal OR did not show significant differences in AMH and FSH levels compared to the control group. This suggests that TAI may not necessarily be associated with a decrease in OR across all patients with TAI. The underlying mechanisms explaining the reduction in OR in some individuals with TAI remain unclear, and further investigation is needed to determine whether this is related to the duration of TAI or potentially to genetic factors.

Our findings are consistent with those of Safarian et al., who reported a significantly lower proportion of MII oocytes in women with TAI after ovarian stimulation. Additionally, they found that the number of MII oocytes was inversely related to the concentration of TPOAbs in the follicular fluid [12]. This suggests that ATAbs in the follicular fluid could contribute to an immunological imbalance during COH, potentially impairing folliculogenesis and oocyte maturation. In our study, although the TAI1 and TAI2 subgroups significantly differed in the mean number of aspirated oocytes following ovarian stimulation, primarily due to differences in OR, no significant differences were found in other key parameters, including the mean percentage of MII oocytes, mean FR, or mean percentage of cleavage stage embryos and cleavage rate. These findings highlight the impact of TAI, since poorer COH outcomes were observed in both TAI subgroups, independent of OR. This supports the hypothesis that an autoimmune process linked to thyroid autoimmunity may impair ovarian gametogenesis despite normal OR. Although a reduced OR worsens COH outcomes independently of TAI, our study confirms that the presence of TAI further impairs COH outcomes, regardless of OR, which is consistent with the findings of Magri et al. [16].

Previous publications on autoimmune thyroid disorders have shown that the presence of ATAbs in serum interferes with ovarian function during stimulation, disrupting the endocrine interplay between the thyroid and ovaries [12,16,22]. Monteleone et al. suggested that TPOAbs may target their antigen directly at the ovarian level, potentially compromising oocyte quality during ovarian stimulation with gonadotropins [22]. Additionally, Miko et al. observed that women with TAI experience an immune imbalance during COH, reflected by an increased ratio of NK and NKT cells. These changes may enhance natural cytotoxicity in TAI-positive women, further impairing normal follicular development [36]. Moreover, Huang et al. demonstrated that TAI-related immunological changes in the follicle involve a chemokine-driven inflammatory cascade, with elevated levels of IFNγ-dependent chemokines (CXCL9, CXCL10, CXCL11), which could help explain the impaired ovarian outcomes observed in our study [37].

As a consequence of impaired ovarian response in TAI, we expected and confirmed that embryo development in culture is significantly impaired in both subgroups of women with TAI. Also, lower percentages of cleavage-stage embryos and blastocysts and reduced cleavage and blastocyst rates were evident. Interestingly, no significant difference in embryo development was observed between the TAI subgroups on Day 3 post-fertilization. Weghofer et al. investigated embryo quality in euthyroid women with low OR treated with IVF and observed comparable embryo quality among women with low–normal or high–normal TSH concentrations (cut-off value of 2.5 mIU/L) [10]. However, poorer embryo quality was observed with positive TPOAbs and TSH ≤ 2.5 mIU/L. Our study did not specifically investigate the effects of TSH levels on embryo development, but focused on OR and thyroid autoimmunity in the euthyroid state. Based on our findings, we propose that ATAbs and an unfavorable immunological milieu during oocyte maturation significantly affect embryo development, potentially impairing the early developmental processes. A retrospective cohort study by Andrisani et al. also found reduced embryo quality in women with at least one positive thyroid autoantibody, which is consistent with our results [11].

Our research demonstrates that TAI is associated with poorer COH and embryo development outcomes independent of OR. Recent work by Herman et al. also found that TAI negatively affected the number of retrieved oocytes and the FR following ICSI in patients younger than 35 years, but not in older patients. This suggests that, while OR may not yet be visibly impaired, TAI could influence the trajectory of ovarian aging [38].

Given the documented overlap between thyroid and ovarian antigens, we hypothesize that the autoimmune process associated with TAI may contribute to the poorer ART outcomes observed in these women [2,4,22,38].

A potential limitation of this study is that all data were derived from a single fertility clinic, which may limit the generalizability of the findings to broader populations. Additionally, participants were classified as TAI-positive or TAI-negative based on predefined cut-off values, and the influence of thyroid autoimmunity was assessed by simultaneously considering both TPOAbs and TgAbs. It is possible that the levels and specific types of antibodies may differentially affect ART outcomes. Moreover, the duration of TAI among participants was not known, which may also influence reproductive outcomes and represents an unaccounted variable in our analysis.

Therefore, our findings suggest that ATAbs play a crucial role in altering the local ovarian endocrine environment despite normal thyroid function and ovarian reserve. The autoimmune thyroid disorder may disrupt oocyte maturation and fertilization, leading to suboptimal ART outcomes. These findings are consistent with previous studies indicating that thyroid autoimmunity negatively affects ovarian function and the overall success of fertility treatments [10,11,12,13,14,15,16,29,30,38]. Further research is needed to fully understand the underlying mechanisms and identify potential interventions to mitigate these effects in women with TAI undergoing ART. A deeper understanding of the pathophysiology of TAI and its impact on fertility and ART outcomes may lead to the development of novel and individualized therapeutic strategies that enhance the success of ART treatments in TAI-positive patients.

## 5. Conclusions

Although the role of TAI in natural conception and its impact on ART outcomes remain subjects of ongoing debate, the findings of our study support the opinion that autoimmune thyroid disease reflects a broader immune dysregulation that disrupts key ovarian processes, including oocyte maturation, folliculogenesis, and embryogenesis. This disruption is evident in the reduced percentage of mature MII oocytes, fertilization rate, and overall embryo development, all of which contribute to the negative influence of TAI on ART outcomes. Our research indicates that TAI is an independent factor that impairs ART success, regardless of ovarian reserve. Further investigation into the endocrinological and reproductive mechanisms underlying this phenomenon is essential to better understand and address the complexities of autoimmune thyroid disease in the context of fertility.

## Figures and Tables

**Table 1:** Basic characteristics of subjects and hormones at the start of COH.

	TAI0	TAI1	TAI2	TAI1 vs. TAI0	TAI2 vs. TAI0	TAI2 vs. TAI1
	(n = 150)	(n = 120)	(n = 71)			
	Mean (±SD)	Mean (±SD)	Mean (±SD)	*p* Value	*p* Value	*p* Value
Female age	35.0 ± 3.2	34.9 ± 3.0	35.9 ± 2.2	0.977	0.065	0.053
TPOAbs (IU/mL)	23.7 ± 10.7	584.8 ± 781.3	414.0 ± 521.4	-	-	0.107
TGAbs (IU/mL)	2.7 ± 1.8	144.6 ± 224.2	167.8 ± 219.9	-	-	0.673
TSH (mIU/L)	1.9 ± 0.7	2.3 ± 1.3	2.1 ± 1.2	0.011 *	0.295	0.613
FSH (IU/L)	7.0 ± 1.4	7.0 ± 2.0	10.1 ± 4.7	0.995	<0.001 *	<0.001 *
LH (IU/L)	6.5 ± 2.0	6.5 ± 2.9	6.4 ± 2.7	0.998	0.881	0.913
E2 (pmol/L)	176.1 ± 69.7	172.0 ± 94.7	195.6 ± 184.8	0.951	0.449	0.338
AMH (ng/mL)	3.0 ± 1.3	3.4 ± 1.7	0.6 ± 0.3	0.112	<0.001 *	<0.001 *

TAI0—control group, TAI1—TAI-positive participants with normal ovarian reserves, TAI2—TAI-positive participants with diminished ovarian reserves; * statistically significant (*p* ≤ 0.05).

**Table 2:** Outcomes of ovarian stimulation and embryo cultures.

	TAI0 (n = 150) Mean/SD	TAI1 (n = 120) Mean/SD	TAI2 (n = 71) Mean/SD	TAI1 vs. TAI0 *p* Value	TAI2 vs. TAI0 *p* Value	TAI2 vs. TAI1 *p* Value
Oocytes retrieved (n)	10.4 ± 4.7	8.8 ± 6.0	3.7 ± 2.5	0.015 *	<0.001 *	<0.001 *
MII oocytes (%)	84.0 ± 14.2	73.8 ± 25.1	73.8 ± 25.1	<0.001 *	0.009 *	0.943
Fertilized oocytes (n)	6.5 ± 2.3	4.5 ± 2.8	2.2 ± 1.8	<0.001 *	<0.001 *	<0.001 *
Fertilization rate (%)	80.8 ± 15.0	70.3 ± 29.3	73.9 ± 33.1	0.002 *	0.139	0.608
Cleavage-stage embryos (3rd day) per MII oocytes (%)	74.4 ± 15.6	63.6 ± 30.2	65.0 ± 35.6	0.002 *	0.037 *	0.933
Blastocyst per MII oocytes (%)	47.1 ± 18.4	26.8 ± 25.8	18.0 ± 30.1	<0.001 *	<0.001 *	0.038 *
Cleavage rate (%)	92.6 ± 11.9	84.3 ± 30.3	79.1 ± 34.9	0.020 *	<0.001 *	0.362
Blastocyst rate (%)	58.2 ± 19.9	35.7 ± 32.5	21.3 ± 34.7	<0.001 *	<0.001 *	0.002 *

TAI0—control group, TAI1—TAI-positive participants with normal ovarian reserves, TAI2—TAI-positive participants with diminished ovarian reserves; * statistically significant (*p* ≤ 0.05).

## Data Availability

The original contributions presented in this study are included in the article. The raw data supporting the conclusions of this article will be made available by the authors on request.

## Abbreviations

The following abbreviations are used in this manuscript:

AMH	Anti-Mullerian hormone
ART	Assisted Reproductive Technology
COH	Controlled Ovarian Hyperstimulation
E2	Estradiol
FR	Fertilization Rate
FSH	Follicle-Stimulating Hormone
IVF	In vitro Fertilization
ICSI	Intracytoplasmic Sperm Injection
LH	Luteinizing Hormone
OR	Ovarian Reserve
SD	Standard Deviation
TAI	Thyroid Autoimmunity
TgAbs	Thyroglobulin Antibodies
TPOAbs	Thyroperoxidase Antibodies
TSH	Thyrotropin
